# Editorial: Data science and health economics in precision public health

**DOI:** 10.3389/fpubh.2022.960282

**Published:** 2022-12-06

**Authors:** Michael A. Talias, Demetris Lamnisos, Alexandros Heraclides

**Affiliations:** ^1^Healthcare Management Postgraduate Program, School of Economics and Management, Open University of Cyprus, Latsia, Cyprus; ^2^Department of Health Sciences, European University Cyprus, Engomi, Cyprus

**Keywords:** health economics, health data science, decision support systems, precision public health, spatial epidemiology

## Overview of precision public health

Advances in computational and data sciences, such as big data approaches, along with engineering innovations, such as geographic information system (GIS) and artificial intelligence (AI) technologies, have greatly enhanced our capabilities for data management, integration, and visualization (1–3). These scientific developments have prompted demands for more comprehensive and coherent but, most importantly, tailored and targeted strategies to address fundamental issues in public health (4, 5). Combining these novel approaches with more traditional health determinants such as lifestyle, socioeconomic, cultural, and environmental factors has resulted in an exciting new field in the health sciences, Precision Public Health (6).

Theory, methods, and models from AI and data science are already changing the public health landscape in community settings (7) and have already shown promising results in multiple applications in public health, including geocoding health data (8), digital public health (9), predictive modeling and decision support (10), and mobile health (11). Overall, Precision Public Health utilizes tools and methods from the above technologies to extract health and non-health data at different levels of granularity, harmonize and integrate information about populations and communities to tailor cost-effective interventions for specific population groups, improving people's health. The overarching goal of Precision Public Health is to provide the proper intervention to the right population at the right time (12).

## Data science in the context of precision public health

Data Science is an interdisciplinary field that is beginning to revolutionize healthcare and public health. This is due to the Digital Revolution that acknowledges the power of digital information and the increasing ability to integrate technologies (13). Novel technological advancements permit the collection and integration of a vast amount of complex and heterogeneous types of data from an increasing array of sources such as genome sequences, social media, satellite remote sensing, earth-observation technologies, electronic health records, Global Positioning System (GPS)-enabled devices, personal sensors, and smartphone application (14). Additionally, there is a considerable decrease in computational costs and abundant storage, processing power, and network connectivity (13). These novel data sources provide the potential to integrate cost-effective and more accurate methods for measuring disease, pathogens, exposomes (the non-genetic exposures experienced over the lifespan), and behaviors that allow better assessment of population health and health determinants (8).

Data Science aims to develop processes and systems to integrate those different data sources, visualize them effectively, and combine advanced analytic methods from several scientific fields such as AI, machine learning, statistics, and high-performance computing to enhance decision-making and develop novel applications in Public Health. In particular, decision-making involves guiding interventions and tailoring public health policies and health promotion programmes, improving early detection of pathogens and infectious disease outbreaks, and enhancing public health surveillance (8). In terms of novel Public Health applications, geospatial applications and predictive modeling techniques identify populations at high risk for a disease, link characteristics of the environment to health changes, explore small area socio-economic inequalities in health, identify and evaluate suspected clusters, and map the risk of diseases.

The development of intelligible, transparent data analytic methods that can be communicated to public health practitioners and easily updated in the face of new data and human judgment will be critical to precision public health (15, 16). Finally, as Mooney and Pejavar stated, the rapid adoption and success of precision public health may depend on how the public health community can embrace a specialized, team science model in training and practice (1).

## Health economics in the context of precision public health

The aim of health Economics is to determine and propose the most efficient means a society allocates its resources for healthcare, disease prevention, and health promotion at the population level (17). In an attempt to address these needs, Health Economic Outcomes Research (HEOR) aims to identify alterations and introductions in the health system (both healthcare and public health) which maximize their beneficial impacts on population health with the minimum possible resource utilization, i.e., ideal efficiency and cost-effectiveness (18).

The concept of Precision HEOR has been recently proposed (18) to optimize the efficiency and cost-effectiveness of health services and systems *via* approaches and sciences, such as big data and machine learning, as described in the previous sub-section. This approach is anticipated to lead to optimized and tailored interventions for specific population groups, lowering costs by avoiding wasting resources resulting from suboptimal treatments, inefficient preventive programmes and other prodigal approaches.

In this respect, AI and machine learning are anticipated to utilize real-world health data on whole populations and specific population subgroups, generated by practical data approaches, informing and enabling the device of more cost-effective and targeted public health services and programmes (19, 20). Additionally, such approaches can be utilized for assessing whether specific public health interventions, which overall might be inferior to other alternative interventions, might be beneficial for particular population subgroups (18).

Additionally, stream approaches involving dynamic model data input methods can incorporate health data streams at the population level as it is made available, increasing direct and timely applicability to those with immediate health needs (21).

Finally, improved cost-effectiveness quickly disregards health inequalities (22). The approaches described so far have the potential to address health inequalities simultaneously by improving the efficiency and cost-effectiveness of public health interventions. This “tailoring” of public health interventions and services to population groups in need, including minority and disadvantaged groups, promises to be invaluable for narrowing the socioeconomic gap in health.

## RT articles

The ten papers in the current RT (Data Science and Health Economics in Precision Public Health) can be grouped into the following broad and non-exclusive categories: Time series and dynamic models (Sun and Yi; Wei et al.; Zhou et al.), Health Inequalities (Peng and Ren; Xin and Ren; Xu et al.), Internet and Social media for health promotion (Han and Zhao; Tchuenche et al.) and Economic evaluations of health (Feng et al.; Lee et al.).

## Conclusion and future steps

Precision Public Health approaches aim to effectively and efficiently implement comprehensive, targeted, and increasingly tailored strategies to address contemporary public health challenges.

Despite the different Public Health fields incorporated in such approaches (e.g., Health Economics and Data Science in this case), there is a relative lack of multidisciplinarity in the design and application of such programmes, as also apparent in the current Research Topic. Incorporation of advances in computational and data sciences (e.g., big data, GIS, AI, and genomics) in more traditional Public Health disciplines (e.g., Health Economics, Health Promotion, Health Protection, Health Inequalities) is anticipated to benefit these disciplines greatly and set the foundations for Public Health practice of the future.

## Author contributions

All authors listed have made a substantial, direct, and intellectual contribution to the work and approved it for publication.

## Conflict of interest

The authors declare that the research was conducted in the absence of any commercial or financial relationships that could be construed as a potential conflict of interest.

## Publisher's note

All claims expressed in this article are solely those of the authors and do not necessarily represent those of their affiliated organizations, or those of the publisher, the editors and the reviewers. Any product that may be evaluated in this article, or claim that may be made by its manufacturer, is not guaranteed or endorsed by the publisher.
